# Apple Polyphenol Mitigates Diabetic Nephropathy via Attenuating Renal Dysfunction with Antioxidation in Streptozotocin-Induced Diabetic Rats

**DOI:** 10.3390/antiox14020130

**Published:** 2025-01-23

**Authors:** Chieh-Yu Wang, Dai-Lin Wu, Meng-Hsun Yu, Chih-Ying Wang, Hsin-Wen Liang, Huei-Jane Lee

**Affiliations:** 1 School of Medicine, Chung Shan Medical University, Taichung 40201, Taiwan; edwina8881@gmail.com (C.-Y.W.); s1001149@gm.csmu.edu.tw (D.-L.W.); 2 Department of Nutrition, Chung Shan Medical University, Taichung 40201, Taiwan; ymh0522@csmu.edu.tw; 3 Department of Nutrition, Chung Shan Medical University Hospital, Taichung 40201, Taiwan; 4 Department of Biochemistry, School of Medicine, Chung Shan Medical University, Taichung 40201, Taiwan; tina0105kimo@yahoo.com.tw; 5 Department of Medical Research, Chung Shan Medical University Hospital, Taichung 40201, Taiwan; vjt8324@gmail.com; 6 Department of Clinical Laboratory, Chung Shan Medical University Hospital, Taichung 40201, Taiwan

**Keywords:** diabetic nephropathy, reactive oxygen species, apple polyphenol

## Abstract

Diabetic nephropathy (DN) is a major cause of morbidity and mortality among patients with diabetes mellitus (DM). Studies have highlighted the critical role of reactive oxygen species (ROS) in the pathogenesis of DM and its complications. Apple polyphenol (AP) has demonstrated antioxidant properties in various models. In this study, we investigated the effects of AP on DN in a rat model. Type 1 diabetes was induced in Sprague–Dawley rats via a single intraperitoneal injection of streptozotocin (65 mg/kg) (*n* = 8). Rats with blood glucose levels exceeding 250 mg/dL were treated with AP at dosages of 0.5%, 1%, or 2% (*w*/*w*) in drinking water for 10 weeks. AP administration significantly improved early-stage DN markers, including reductions in the blood urea nitrogen-to-creatinine ratio and the urinary albumin-to-creatinine ratio (ACR), in a dose-dependent manner. AP treatment also significantly lowered blood triglyceride levels and reduced lipid peroxidation in kidney tissues. Histological analysis revealed that AP attenuated renal hydropic change, reduced glomerular basement membrane thickening, and restored mitochondrial morphology in diabetic rats. Additionally, the upregulation of transforming growth factor-beta (TGF-β) observed in the diabetic kidney was attenuated by AP treatment. In H_2_O_2_-stimulated rat mesangial cells, AP reduced ROS levels, accompanied by a reduction in TGF-β expression. These findings suggest that AP exerts protective effects against DN by improving renal function and mitigating oxidative stress, indicating its potential as a nutraceutical supplement for slowing DN progression.

## 1. Introduction

Diabetic nephropathy (DN) is a major complication of diabetes mellitus (DM) that significantly contributes to morbidity and mortality in diabetic patients [1]. Characterized by persistent albuminuria (>3 mg/mmol) over a prolonged period (>90 days) and/or a progressive decline in estimated glomerular filtration rate (eGFR < 60 mL/min/1.73 m^2^), DN can lead to end-stage renal disease (ESRD) [2]. The pathogenesis of DN is complex, with hyperglycemia-induced oxidative stress playing a pivotal role. Hyperglycemia activates multiple pathways, including the polyol pathway, protein kinase C, hexosamine biosynthesis pathway, and advanced glycation end product formation, leading to an increased generation of reactive oxygen species (ROS) [1]. This imbalance between oxidants and antioxidants results in excessive ROS accumulation, which contributes to inflammation, fibrosis, and structural alterations in renal tissues, such as glomerular hypertrophy, mesangial matrix expansion, and thickening of the glomerular basement membrane (GBM) [3].

Elevated levels of malondialdehyde (MDA) and protein carbonyls, markers of oxidative stress, exacerbate DN progression [4]. The antioxidant defense system, including superoxide dismutase (SOD), glutathione peroxidase (GSH-Px), catalase, and glutathione reductase, is activated in response to increased reactive oxygen species (ROS) accumulation [5]. However, hyperglycemia further promotes pro-inflammatory and profibrotic responses, leading to the upregulation of tumor necrosis factor-α (TNF-α) and transforming growth factor-β (TGF-β) [6]. These factors contribute to mesangial and endothelial cell damage, ultimately disrupting the glomerular filtration barrier [7]. Furthermore, in the early stages of DN, conditions such as hyperglycemia, dyslipidemia, hypertension, and the accumulation of advanced glycation end products stimulate glomerular mesangial cells. This stimulation induces mesangial cell hyperplasia, contributing to renal microvascular damage and fibrosis. Consequently, mesangial cells play a pivotal role in the onset and progression of DN. Inhibiting mesangial cell proliferation represents a critical therapeutic strategy for mitigating the progression of DN [8].

Given the pivotal role of oxidative stress in the progression of DN, antioxidant therapy has emerged as a promising strategy for attenuating disease progression. Natural compounds with antioxidant properties have been investigated for their potential to mitigate oxidative damage and protect the kidneys. Quercetin, in streptozotocin (STZ)-induced DN rats, has demonstrated efficacy in reducing blood urea nitrogen and serum creatinine levels, increasing the expression of nephrin and podocin while decreasing desmin, and ameliorating podocyte effacement [9]. Low-dose quercetin or quercetin liposomes can attenuate renal pathological changes, including glomerular volume atrophy, excessive extracellular matrix and glycogen deposition, basement membrane thickening, and tubulointerstitial fibrosis [10,11]. The literature has summarized the potential of plant polyphenols as dietary antioxidants to protect against diabetes, cancers, and cardiovascular diseases [12,13,14]. Among these polyphenols, apple polyphenol (AP) has garnered attention due to its diverse biological activities, including anti-cancer [15], cardioprotective [16], and antioxidant effects [17]. Among AP, a diverse array of polyphenolic compounds, including flavonols, flavanols, and procyanidins, significantly contribute to its antioxidant capacity. These compounds exhibit a remarkable ability to scavenge free radicals and enhance overall antioxidant activity, often surpassing the efficacy of common antioxidants such as vitamin C [18]. Furthermore, recent studies have demonstrated that AP activates the Nrf2/Keap1 signaling pathway, a critical regulator of cellular antioxidant defense mechanisms [19]. Previous studies have demonstrated AP can decrease inflammatory and profibrotic proteins in a rat model of unilateral ureteral obstruction [20]. Despite these well-documented benefits, the therapeutic potential of AP in DN remains largely unexplored.

This study aims to investigate the protective effects of AP on DN in a STZ-induced type 1 diabetes model in Sprague–Dawley (SD) rats. We hypothesize that AP can attenuate the progression of DN by reducing oxidative stress, improving renal function, and preventing structural damage to the kidneys, which is specific to glomerular mesangial cells. Through biochemical, histological, and oxidative stress assessments, we seek to evaluate the potential of AP in managing DN.

## 2. Materials and Methods

### 2.1. Apple Polyphenols (APs)

APs (Applephenon^®^) were obtained from Asahi Co. (Tokyo, Japan). As described in previous reports [21,22], Applephenon^®^ is prepared from unripe apples (Malus pumila cv. Fuji). Studies have demonstrated that unripe apples contain higher concentrations of polyphenols than ripe apples, including 63.8% procyanidins composed of 11.1% dimers, 12.3% trimers, 8.7% tetramers, 5.9% pentamers, 4.9% hexamers, and 20.9% other polymers. Additionally, Applephenon^®^ contains 12.4% flavan-3-ols (monomers), 6.5% other flavonoids, and 10.8% non-flavonoids [23,24].

### 2.2. Animals

**Figure 1:**
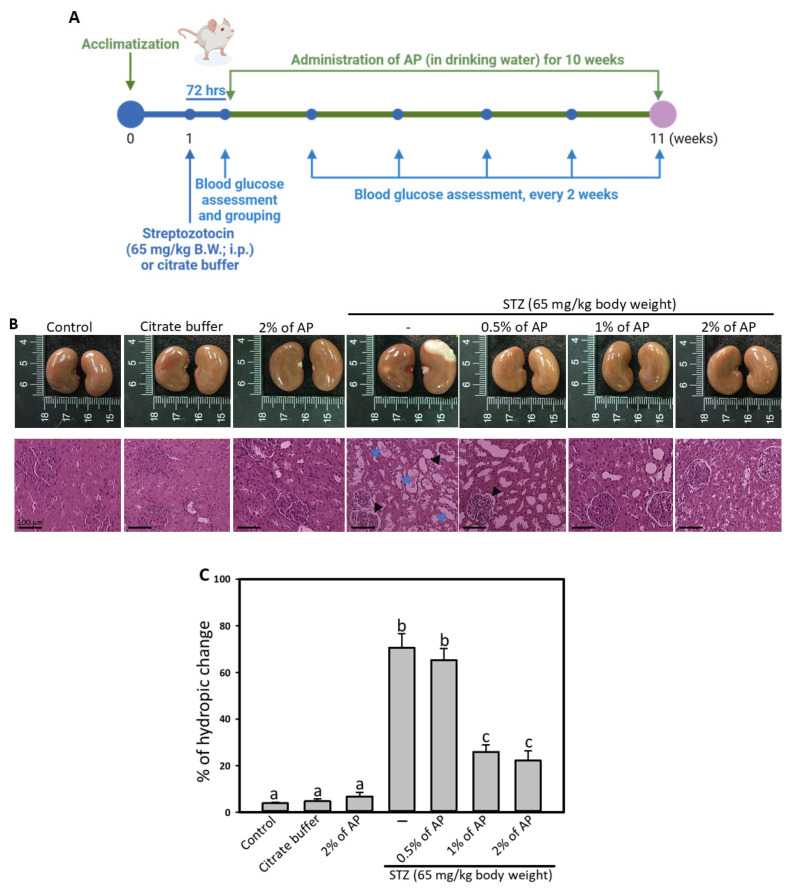
Effect of AP on kidney damage in diabetic rats. (**A**) experimental design. SD rats were assigned into the following groups: control; citrate buffer; 2% AP only; 65 mg/kg of STZ; STZ and administered with 0.5%, 1%, or 2% of AP. After being treated with AP for 10 weeks, the kidneys were removed, and the morphology is shown in (**B**), upper panels; H&E stains were performed and the histological changes are shown in (**B**), lower panels. Blue arrowheads point the hydropic changes, shown as a pale and swollen change of the proximal convoluted tubules; black arrowheads represent the GBM, shown as a white edge surrounding the glomeruli. Scale bar: 100 mm. (**C**), quantification of the rate of hydropic change; results were represented as mean ± SD; values not sharing a common letter in the same row are significantly different (*p* < 0.05).

Four-week-old male SD rats obtained from BioLASCO Taiwan Co., Ltd. (Yilan County, Taiwan) were housed in a standard animal laboratory with a controlled temperature of 22 °C, humidity of 55%, access to drinking water, standard rat feed, and a 12-h light/dark cycle. Following the protocols of the Animal Models of Diabetic Complications Consortium (AMDCC), animals were fasted for 12 h and then administered a single intraperitoneal (i.p.) injection of streptozotocin (STZ, Sigma-Aldrich Corp., St. Louis, MO, USA) at a dose of 65 mg/kg dissolved in 0.1 M citrate buffer (pH 4.5). After 72 h, tail vein blood glucose levels were measured after an overnight fast. Diabetes was diagnosed if blood glucose concentrations exceeded 250 mg/dL. Building upon previous studies that utilized a feeding model to investigate the effects of plant polyphenols on delaying DN [25], the administration mode was modified to drinking in the present study. Preliminary experimental results demonstrated that an 8-week administration of 1% AP significantly reduced the renal function marker urine microalbumin-to-creatinine ratio (ACR). Urine ACR represents the ratio of urinary microalbumin to creatinine. Clinically, it serves as a crucial indicator for detecting early kidney injury, providing an important measure of kidney function. Based on these findings, diabetic rats (*n* = 8) were then randomly divided into four groups: STZ-induced diabetic control and three groups treated with AP diluted in drinking water at concentrations of 0.5%, 1%, and 2%, respectively. Three control groups were included: a normal control, a citrate buffer control (which received a single i.p. injection of 0.1 M citrate buffer), and a 2% AP-alone control (shown as Figure 1A). All rats were provided with standardized food (Purina Laboratory Chow, Purina Mills, Inc., St. Paul, MN, USA) and water ad libitum. Blood glucose levels were monitored every two weeks via tail vein blood sampling. Urine was collected and analyzed at the tenth week, after which the animals were sacrificed for blood and kidney analysis. The animal experimental protocol used in this study was approved by the Institutional Animal Care and Use Committee of Chung Shan Medical University (IACUC Approval No. 700, CSMC), Taichung, Taiwan.

### 2.3. Biochemical Analysis

Blood glucose, albumin, blood urea nitrogen (BUN), and creatinine levels were determined using enzymatic colorimetric methods with an automated analyzer (Olympus AU2700, Olympus Co., Tokyo, Japan). Concentrations of total cholesterol and triglyceride were measured using enzymatic colorimetric methods with commercial kits (Boehringer Mannheim, Mannheim, Germany).

### 2.4. Pathological Histology of Kidney

Following euthanasia, kidneys were immediately excised and fixed in 10% buffered formaldehyde. Subsequently, the tissues were processed for histological examination using conventional techniques and stained with hematoxylin and eosin. Microscopic evaluation of kidney lesions was conducted to assess hydropic degeneration. The severity of kidney lesions was quantified by examining ten randomly selected high-power fields (×200) under a light microscope. The percentage of lesion areas in kidney specimens was determined using Image Pro Plus 4.0 software.

For electron microscopy analysis (JEM-1230 Electron Microscope, JEOL, Tokyo, Japan), the initial processing steps were followed. Brightness and contrast parameters were adjusted, and regions of interest with the desired brightness were selected. Morphometry measurements were performed after noise and disturbance filtering. The thickness of the glomerular basement membrane was quantified by examining ten randomly selected fields.

### 2.5. Thiobarbituric Acid-Reacting Substances (TBARSs)

Lipid peroxidation was assessed by measuring thiobarbituric acid reactive substances (TBARSs). Kidney tissue specimens (0.5 g) were homogenized in 5 mL of 50 mM phosphate buffer (pH 7.4) and centrifuged (12,000× *g*) for 30 min to obtain the supernatant homogenate. Protein content in the supernatant was quantified using the Bio-Rad protein assay reagent with bovine serum albumin as a standard. The calibration curve ranged from 0 to 400 μg/mL, with a correlation coefficient (r^2^) of 0.9921. A 0.3 mL aliquot of homogenate was mixed with 0.3 mL of thiobarbituric acid (TBA, 1% in 0.3% NaOH) and reacted for 40 min at 95 °C in the dark. The resulting samples were analyzed using a spectrophotofluorimeter (Hitachi F2000, Tokyo, Japan) with an excitation wavelength of 532 nm and an emission wavelength of 600 nm. A calibration curve was constructed using malondialdehyde (MDA) standards ranging from 0 to 50 nmol (r^2^ = 0.9915). TBARS concentrations were expressed as equivalents of MDA in nanomoles per milligram of protein.

### 2.6. Immunohistochemistry

Kidneys were sectioned into moderate-sized pieces and immersed in 70% alcohol for 24 h to fix the tissue. Subsequently, the samples were dehydrated in a series of ascending alcohol concentrations, deparaffinized with xylene, and embedded in paraffin. The tissue was then sectioned into 5 µm thick sections, placed on slides, and heated at 38 °C for extension. After dewaxing, the sections were boiled in citrate buffer (0.01 M, pH 6.0) for ten minutes and rinsed with secondary water for five minutes. Dual Endogenous Enzyme Block was applied for five minutes to block endogenous enzymes. The sections were then washed with phosphate-buffered saline containing Tween-20 (PBST) and incubated with a TGF-β antibody (1:200, ab215715, Abcam Co., Cambridge, UK) for three hours. Following a PBST wash, labeled polymer-horseradish peroxidase was applied for 30 min. After another PBST wash, the reaction products were visualized using 3,3′-diaminobenzidine (DAB, Thermo Fisher Scientific Inc., Waltham, MA, USA) in a buffer substrate. The tissue was counterstained with hematoxylin for 2 min and dehydrated. The quantification of TGF-β was performed by examining ten randomly selected high-power fields (×200) under a light microscope.

### 2.7. Glutathione S-Transferase (GST) Activity Assay

Kidney tissue obtained from the rats was homogenized in a lysis buffer (150 mM NaCl, 1% Nonidet P-40, 0.5% deoxycholic acid, 0.1% SDS, and 50 mM Tris base, pH 7.5), and centrifuged at 12,000× *g* for 30 min at 4 °C. For the GST activity assay, 40 μL of the supernatant was added to a reaction buffer containing 50 mM potassium phosphate buffer (pH 6.5), 1 mM GSH, and 1 mM CDNB in absolute ethanol, and incubated at 25 °C. Absorbance at 340 nm was recorded at 1-min intervals over 5 min. The change in absorbance within the linear range was calculated as ΔA340/min = [A340 (final) − A340 (initial)]/reaction time (min), with the blank ΔA340/min subtracted from the sample ΔA340/min. GST-specific activity was calculated using the formula [ΔA340/min × reaction volume (mL)]/[εmM × sample volume (mL) × path length] = μmol/mL/min, where εmM is the extinction coefficient for the CDNB conjugate at 340 nm [26].

For the GST-α activity assay, 40 μL of supernatant was mixed with a reaction buffer containing 50 mM potassium phosphate buffer (pH 6.5), 1 mM GSH, and 1 mM NBD-Cl, and incubated at 25 °C. Absorbance at 419 nm was recorded at 0.5-min intervals over 3 min, and the activity was calculated using the formula above.

For the GST-π activity assay, 40 μL of supernatant was added to a reaction buffer with 50 mM potassium phosphate buffer (pH 6.5), 1 mM GSH, and 0.2 mM ethacrynic acid, and incubated at 25 °C. Absorbance at 270 nm was recorded at 0.5-min intervals for 3 min, and activity was calculated as described above.

### 2.8. Cell Culture

Rat mesangial cells (RMCs), obtained from the Bioresource Collection and Research Center (BCRC, Hsinchu, Taiwan), were cultured in Dulbecco’s Modified Eagle Medium supplemented with 15% fetal bovine serum, 1% penicillin-streptomycin, 1% L-glutamine (Gibco Laboratory, Gaithersburg, MD, USA), and 0.4 mg/mL G418 (Sigma-Aldrich Ltd., St. Louis, MO, USA). Cultures were maintained in an incubator at 37 °C with 5% CO_2_ and 95% O_2_.

### 2.9. MTT Assay

Cells were seeded at a density of 1 × 10^5^ cells/mL in 24-well plates and incubated for 16 h. Following this, cells were treated with various concentrations of AP (0.03, 0.05, 0.1, 0.3, 0.5, 0.8, and 1 mg/mL) for 24 h. The culture medium was then removed, and MTT [3-(4,5-dimethylthiazol-2-yl)-2,5-diphenyltetrazolium bromide] was added to each well at a final concentration of 0.5 mg/mL for a 3-h incubation period. The resulting formazan crystals were solubilized in isopropanol, and absorbance was measured at 563 nm using a spectrophotometer (Hitachi U2001, Hitachi Co., Tokyo, Japan). Absorbance values were used to calculate viable cell numbers, with results from three independent experiments expressed as percentages normalized to the control group.

### 2.10. Measurement of ROS Production

ROS activity was measured using the Cellular ROS Detection Assay Kit (Abcam Co., Ltd., Cambridge, UK). Briefly, cells were seeded at a density of 1 × 10^5^ cells/mL in a 10 cm petri dish. Upon adherence, cells were treated with 500 mM of hydrogen peroxide (H_2_O_2_) with or without 0.08 mg/mL AP for 24 h. After removing medium, DCFDA/H_2_DCFDA was deacetylated by cellular esterases into a non-fluorescent compound, which was subsequently oxidized by ROS to form 2′,7′-dichlorofluorescein (DCF). DCF fluorescence was measured via FACScan cytometry (BD Biosciences, San Jose, CA, USA). Signals from 10,000 cells were acquired, and fluorescence intensities were quantified using CellQuest Pro software (version 5.1, BD Biosciences, San Jose, CA, USA).

### 2.11. Measurement of Mitochondrial Membrane Potential (ΔΨm)

Following the appropriate treatments, cells were gently dislodged and incubated with 2 μM 5,5′,6,6′-tetrachloro-1,1′,3,3′-tetraethylbenzimidazolylcarbocyanine iodide (JC-1, Thermo Fisher Scientific Inc., Waltham, MA, USA) dye in 1 mL PBS at 37 °C for 15 min. The cells were then immediately examined and photographed using a fluorescence microscope, and the observations were compared with images captured under bright-field microscopy at 200× magnification. Ten randomly selected areas calculated the JC-1 density for the quantification using ImageJ software (deepImageJ v.2.1.0) (National Institutes of Health, Bethesda, MD, USA).

### 2.12. Immunoblot

RMCs were harvested and suspended in a lysis buffer (50 mM Tris, 5 mM EDTA, 150 mM NaCl, 1% NP40, 0.5% deoxycholic acid, 1 mM sodium orthovanadate, 81 μg/mL aprotinin, 170 mg/mL leupeptin, 100 μg/mL PMSF; pH 7.5) for 1 h at 4 °C. The lysates were subsequently centrifuged at 10,000× *g* for 10 min, and the supernatants were collected to obtain the whole-cell extracts. Protein concentration was determined using the Bradford protein assay (Kenlor Industries, Costa Mesa, CA, USA). Equal amounts of protein sample were then subjected to a 12% SDS-polyacrylamide gel electrophoresis to separate, followed by being electro-transferred onto nitrocellulose membranes (Sartorius Co., Goettingen, Germany). The membranes were incubated with anti-TGF-b antibody (1:1000, sc-130348, Santa Cruz Biotechnology, Inc., Dallas, TX, USA). Detection was achieved using Western Blot Chemiluminescence Reagent Plus (Thermo Fisher Scientific Co., Waltham, MA, USA), and chemiluminescent signals were captured and quantified using a digital imaging system (FujiFilm LAS-1000 plus, American Laboratory Trading Co., San Diego, CA, USA).

### 2.13. Statistical Analysis

Statistical analysis of the data was performed using SigmaStat statistical software (version 4.0, Jandel Scientific Software, Corte Madera, CA, USA). Mean differences were evaluated at a 95% confidence level using one-way analysis of variance (ANOVA) with Duncan’s multiple range tests followed by the least significant difference (LSD) post-hoc test to compare various groups. The Student’s *t*-test was used to make pairwise comparisons. All experiments were conducted independently three times, and all data were presented as mean ± standard deviation (SD). Statistical significance was considered at a probability level of *p* < 0.05.

## 3. Results

### 3.1. AP Improved Renal Function in Diabetic Rats

After completing the animal treatment, blood and urine samples were collected to analyze molecules associated with renal function. As shown in Table 1, STZ treatment for 10 weeks reduced body weight to 51.9% compared to the control group, while rats treated with 2% AP showed significant body weight increases of approximately 21.3% compared to the STZ group. STZ treatment increased kidney weight to 11.2% compared to the control group, while rats treated with 1 or 2% AP showed significant kidney weight decreases of approximately 102.5 or 130% compared to the STZ group. However, AP did not reduce STZ-induced elevations in blood glucose or excessive urine output. In conditions of renal impairment, high levels of serum albumin can leak into the urine. The serum albumin level was significantly lower in the STZ group than in the control group. Treatment with 1% AP led to a significant 13.7% increase in serum albumin compared to the STZ group. Current findings indicate that AP treatment does not significantly improve BUN or blood creatinine levels in diabetic animals. Since the ratio of BUN to creatinine is useful for assessing early kidney damage [27], we calculated this ratio and found that AP reduced the STZ-induced BUN-to-creatinine ratio in a dose-dependent manner. Early kidney damage may result in microalbuminuria due to decreased glomerular filtration rates, and microalbumin levels can be predictive of DN. At week 10, 24-h urine samples were collected, and analysis showed a 58% increase in urinary microalbumin in the STZ-induced group compared to the control group. Treatment with 1% and 2% AP reduced urinary microalbumin levels by approximately 47% and 48%, respectively. Furthermore, we calculated ACR to evaluate glomerular filtration rates in early DN. The ACR level in the STZ group increased by 95% compared to the control group, while 10 weeks of treatment with 2% AP decreased the ACR level by 68% relative to the STZ group (Table 1). These results indicate that AP treatment can improve renal function in early DN.

### 3.2. AP Mitigated the Histological Alternation of Diabetic Kidneys

Following euthanasia, a marked renal lesion was observed in STZ-induced diabetic rats, while rats treated with AP did not exhibit this renal lesion (Figure 1B, upper panels). Hydropic degeneration of the proximal convoluted tubules, characterized by a swollen and pale renal tubular epithelium, was significantly increased in STZ-treated rats, affecting approximately 70.56% of tubules compared to the control group. In contrast, AP treatment significantly reduced these hydropic changes to approximately 25.96% and 22.16% in the 1% and 2% AP-treated groups, respectively (Figure 1B, lower panels, and Figure 1C).

**Figure 2:**
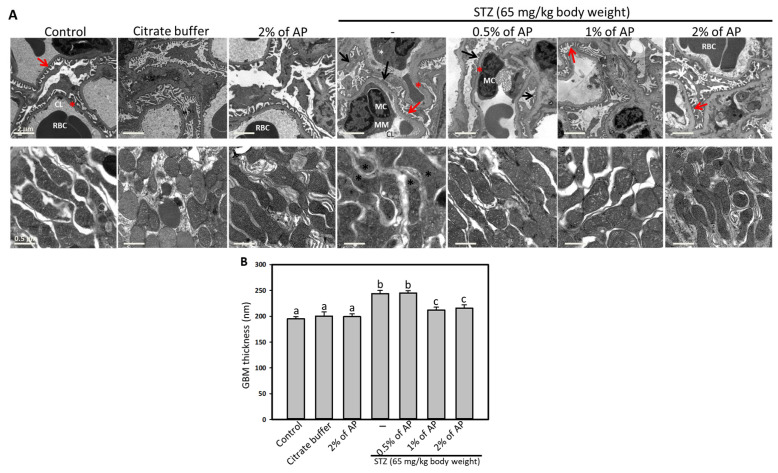
The effect of AP on the glomerulus and mitochondria profile. Electron microscopy analysis was performed to examine the glomerulus and mitochondria in kidney sections from the animals. (**A**), upper panels display the histological analysis of glomeruli. CL, capillary lumen; MC, mesangial cell; MM, mesangial matrix; red arrows indicate the podocyte foot processes; black arrows indicate partial foot process effacement; the red asterisk shows the glomerular basement membrane; scale bar, 2 mm; lower panels display the mitochondrial morphology in the kidney. The black asterisk shows the damaged mitochondria in the kidneys; scale bar, 0.5 mm. (**B**), quantification of the thickness of GBM; results are represented as mean ± SD; values not sharing a common letter in the same row are significantly different (*p* < 0.05).

Structural alterations manifest in the glomerulus during the progression of DN [3]. To assess the effect of AP on the glomerulus, GBM thickness was examined under an electron microscope. As shown in the upper panels of Figure 2A,B, GBM thickness was significantly increased in STZ-treated rats compared to the control group, while GBM thickening was reduced in AP-treated groups, indicating that AP effectively attenuates GBM thickening. Additionally, electron microscopy revealed an expansion of the mesangial matrix in the kidney sections of diabetic rats, which was notably regressed in the AP-treated groups (Figure 2A, upper panels). In diabetic rats, partial foot process effacement observed in kidney sections indicated podocyte impairment, whereas this condition was ameliorated in the group treated with 2% AP (Figure 2A). The morphology and ultrastructure of mitochondria in mesangial and proximal tubule cells were also examined. In diabetic rats, mitochondria exhibited substantial morphological damage, including swelling, membrane disruption, blurred cristae, and loss of the boundary with the intermembrane space, leading to a molded configuration due to elongated and compressed mitochondria in both mesangial and proximal tubule cells. Although some mitochondrial lesions persisted in the AP-treated groups, the original mitochondrial morphology was significantly restored, resembling that observed in the control group (Figure 2A, lower panels). These results suggest that AP treatment ameliorates key histological changes in the diabetic kidney, thereby supporting renal function in diabetic conditions.

### 3.3. Effects of AP on Renal Oxidative Status in STZ-Induced Diabetic Rats

In the control group, the cholesterol level was 61.0 ± 7.0 mg/dL. Treatment with STZ alone led to a marked decrease to 36.0 ± 1.8 mg/dL. Rats treated with 1% or 2% AP exhibited total cholesterol levels of 72.3 ± 25.1 or 67.0 ± 15.1 mg/dL, respectively, which were significantly higher than those in the STZ group and not statistically different from the levels in the control group. AP treatment showed an increase in cholesterol levels in the STZ-treated groups. Blood analyses revealed that triglyceride levels in STZ-induced diabetic rats were elevated 2.23-fold compared to the control group, whereas AP treatment significantly reduced triglyceride levels (Table 2). Elevated triglycerides increase serum fatty acid levels, which can induce lipid peroxidation. TBARS analysis showed a significant 1.69-fold increase in lipid peroxidation in STZ-treated rats relative to controls, while AP administration reduced renal lipid peroxidation. GSTs, major phase II detoxification enzymes found primarily in the cytosol, exhibit peroxidase and isomerase activities that protect cells from oxidative stress-induced death [28]. Following STZ treatment, total renal GST levels in diabetic rats decreased by 35% compared to the control group. However, treatment with 0.5%, 1%, and 2% AP did not significantly restore renal GST levels. GST protein family subtypes are ubiquitous enzymes involved in the detoxification of free radicals [29]. In this study, we also examined the expression of GST-α and GST-π. As indicated in Table 2, GST-α levels were significantly reduced in the STZ-treated group (19.5 ± 3.2 nmole/min/mg protein) compared to the control group (30.5 ± 6.5 nmole/min/mg protein). GST-α is an antioxidant enzyme responsible for detoxifying harmful metabolites under conditions of oxidative stress. While treatment with AP at 1% and 2% concentrations led to slight increases in GST-α levels (18.9 ± 3.1 and 18.8 ± 9.5 nmole/min/mg protein, respectively), these changes were not statistically significant compared to the STZ-treated group, suggesting that AP may have limited effects on GST-α expression in this diabetic model. Similarly, GST-π levels, another isoform involved in antioxidative mechanisms, were also significantly reduced in the STZ group (4.8 ± 0.3 nmole/min/mg protein) compared to controls (5.1 ± 0.7 nmole/min/mg protein). AP treatment did not significantly restore GST-π levels, with the 1% or 2% AP-treated groups showing levels of 4.4 ± 0.8 or 3.1 ± 0.2 nmole/min/mg protein, respectively. This finding indicates that while AP has demonstrated efficacy in improving other markers of oxidative stress, its influence on GST isoforms may be more limited in the current model.

### 3.4. AP Reduced the TGF-β Level in the Kidneys of STZ-Treated Rats

**Figure 3:**
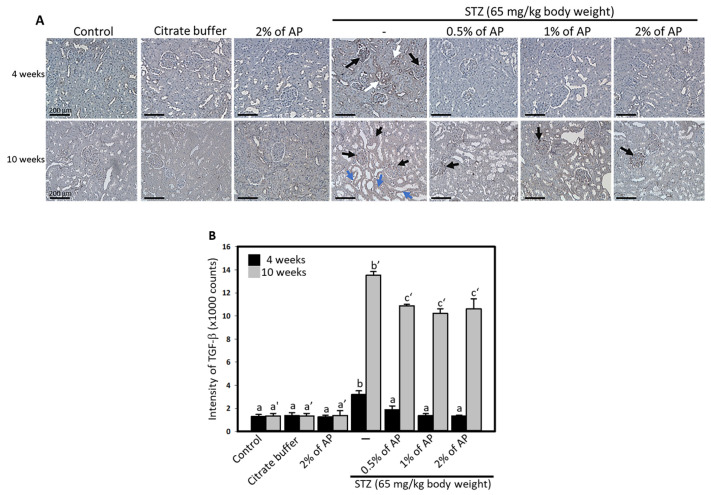
AP reduced TGF-β expression in diabetic kidneys. Immunohistological analysis was performed to evaluate TGF-β expression in rat kidneys. In addition to analyzing kidney samples from animals treated for 10 weeks, the TGF levels were also compared in kidney samples from animals treated for 4 weeks. (**A**), upper panels, 4 weeks; lower panels, 10 weeks. Black arrows point to positive-stained TGF-β in the proximal convoluted tubules. Blue arrows point TGF-β in the glomeruli. Scale bar, 200 mm. (**B**), quantification results showing the expressed intensity of TGF-β. Results are represented as mean ± SD; values not sharing a common letter in the same row are significantly different (*p* < 0.05).

TGF-β is recognized as a central mediator of fibrosis and the accumulation of extracellular matrix (ECM) in both the glomerulus and tubulointerstitial compartments following renal injury [6]. To assess the effects of STZ on the kidney, immunohistochemical staining was conducted to detect TGF-β expression in euthanized rats at week 10. We also conducted a comparative analysis using kidney samples obtained from animals treated with STZ or AP for 4 weeks. As depicted in Figure 3A, TGF-β expression was elevated in both the proximal convoluted tubules and glomeruli of STZ-treated rats at both time points. However, AP treatment significantly reduced TGF-β levels. Quantitative analysis further illustrated the distribution of TGF-β-positive areas in each group (Figure 3B).

### 3.5. AP Reduced Oxidative Stress and Improved Mitochondrial Membrane Potential in H_2_O_2_-Induced RMCs

**Figure 4:**
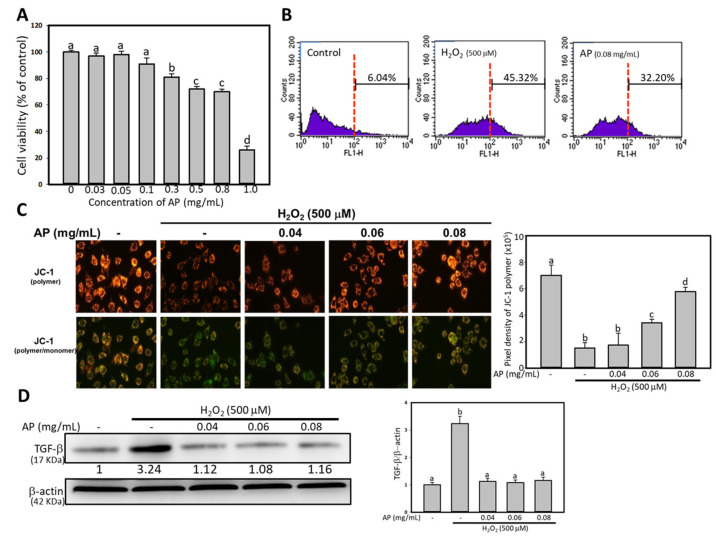
(**A**), cell viability: AP reduced oxidative status/TGF-β and restored mitochondrial function in H_2_O_2_-induced RMCs. RMCs at a concentration of 1 × 10^5^ cells/mL were treated with 500 mM of H_2_O_2_ or co-treated with AP for 24 h. Results from three independent experiments are represented as mean ± SD; values not sharing a common letter in the same row are significantly different (*p* < 0.05); (**B**), the DCF-DA intensity detected by FACScan cytometry: the area to the right of the red dashed line was defined as exhibiting ROS signals; (**C**), JC-1-stained cells were analyzed using fluorescence microscopy with the quantified result. Magnification: ×200; (**D**), the protein level of TGF-b with the quantified results. The quantified results of JC-1 and TGF-b examinations from three independent experiments are represented as mean ± SD; values within the same row that do not share a common letter are significantly different (*p* < 0.05).

The cytotoxic effects of AP on RMCs were assessed using the MTT assay. RMCs were treated with varying concentrations of AP (0.03, 0.05, 0.1, 0.3, 0.5, 0.8, and 1.0 mg/mL) for 24 h. As shown in Figure 4A, AP concentrations above 0.3 mg/mL significantly inhibited RMC proliferation, with an IC_50_ of approximately 0.9 mg/mL. To elucidate the mechanisms of AP in vitro, concentrations of 0.04, 0.06, and 0.08 mg/mL were selected for further analysis. ROS levels in RMCs were measured following treatment with 0.08 mg/mL of AP and H_2_O_2_. As shown in Figure 4B, the mean ROS level in the control group was 6.04%. Treatment with 500 µM H_2_O_2_ elevated the mean ROS level to 45.32%, while 0.1 mg/mL AP reduced ROS levels to 32.0%. JC-1 is a cationic, positively charged fluorescent dye that exhibits potential-dependent accumulation within mitochondria. It is indicated by a fluorescence emission shift from green (monomer) to red (polymer). A decrease in red fluorescence intensity reflects mitochondrial depolarization. JC-1 staining was performed to evaluate the effect of AP on mitochondrial membrane potential. A shift in fluorescence from red to green indicated a disruption of the mitochondrial membrane potential following 500 µM H_2_O_2_ treatment (Figure 4C). Treatment with AP at concentrations of 0.06 and 0.08 mg/mL significantly restored mitochondrial membrane potential (Figure 4C). Figure 4D demonstrates that treatment with 500 µM H_2_O_2_ increased TGF-β protein levels; however, AP treatment at concentrations of 0.04, 0.06, and 0.08 mg/mL significantly reduced TGF-β expression.

## 4. Discussion

DN begins in the early stages of diabetes and represents an irreversible pathological progression. In this study, conducted in both animal and cellular models, results demonstrate that AP can reduce ROS, decrease lipid peroxidation, delay TGF-β expression, mitigate ultrastructural alterations in renal tissues, and improve renal function. This provides an alternative perspective for the use of nutraceuticals in delaying the progression of DN.

STZ induces destruction of pancreatic β-cells, leading to insulin deficiency and the inability to maintain glucose homeostasis. In STZ-induced animal models, type 1 DM (T1DM) is commonly observed. The findings of this study indicate that AP administration does not reduce STZ-induced hyperglycemia. This may be attributed to the irreversible β-cell damage caused by STZ, as AP was administered after the onset of STZ-induced hyperglycemia and thus could not reverse the damage to lower blood glucose levels. Previous studies also demonstrated that in STZ-induced T1DM, neither cilostazol nor polyphenol extracts from Hibiscus sabdariffa Linnaeus were effective in reducing blood glucose levels [25,30,31]. Studies suggest that polyphenols may aid in reducing blood glucose levels and contribute to a decreased risk of type 2 DM (T2DM) [32,33]. In individuals with T2DM, insulin levels may be low, normal, or elevated; however, partial β-cell function is retained, allowing polyphenols to potentially preserve β-cell activity through antioxidative and anti-inflammatory mechanisms, thereby supporting glucose homeostasis. Current evidence suggests that while AP does not mitigate STZ-induced hyperglycemia in T1DM, AP treatment may enhance renal function, reduce histopathological changes, decrease oxidative stress accumulation, and alleviate STZ-induced DN at both the histological and cellular levels.

Previous studies have demonstrated that in diabetic patients, elevated triglyceride levels correlate with increased plasma lipid peroxidation, as indicated by elevated TBARS levels, suggesting that dyslipidemia substantially contributes to oxidative stress in DM [34]. Our current findings indicate that AP significantly reduces triglyceride levels and lipid peroxidation in STZ-treated rats and decreases ROS levels in H_2_O_2_-treated RMCs. The capacity of AP to reduce oxidative stress and ROS may contribute to the mitigation of DN. The progression of DN in T1DM and T2DM involves distinct underlying mechanisms. In T1DM, oxidative stress is a primary driver of basement membrane dysfunction, primarily through ROS generation and the resulting disruption of cellular signaling pathways [3]. Mitochondrial dysfunction may lead to increased oxidative stress. In DN, hyperglycemia induces increased ROS production from sources such as NADPH oxidases and mitochondrial dysfunction, leading to pathological changes, including glomerulosclerosis and tubulointerstitial fibrosis [35,36,37]. Conversely, in T2DM, insulin resistance or oxidative stress contributes to BM dysfunction by impairing insulin signaling in podocytes, resulting in structural damage, proteinuria, and glomerulosclerosis, which exacerbates DN and the progression of chronic kidney disease. Although the etiology is varied, both types of DM exhibit mesangial expansion and podocyte alterations, contributing to BM dysfunction and the progression of DN [38]. In the context of the short-term effects of a 10-week treatment, the mechanism by which AP improves renal dysfunction in T1DM may be partially attributed to its antioxidative properties. However, considering the progressive nature of DN, a 10-week treatment period of AP may be insufficient to achieve a significant reduction in each parameter. Therefore, future studies and applications may require prolonged and continuous administration of AP to effectively observe its therapeutic effects.

In addition to assessing lipid peroxidation, GST levels were measured in kidney specimens; however, no significant changes were observed following treatment with AP. This phenomenon may be attributed to several interrelated factors, including impaired synthesis and increased degradation of GSH under diabetic conditions, likely due to sustained hyperglycemia, as discussed previously. Studies indicate that DM significantly reduces the activity of γ-glutamylcysteine synthetase, an enzyme essential for GSH synthesis, leading to decreased GSH concentrations in various tissues, including the liver and erythrocytes [39,40]. Furthermore, other studies have demonstrated that while antioxidants such as N-acetylcysteine and taurine can partially improve GSH levels, they do not fully restore GSH synthesis under the oxidative stress associated with DM, resulting in persistent GSH depletion rather than effective replenishment [40]. Thus, the minimal changes observed in GSH levels may be attributed to oxidative stress resulting from STZ-induced hyperglycemia due to irreversible β-cell damage, thereby limiting the efficacy of antioxidant treatment in modulating GSH levels. On the other hand, the Applephenon^®^ used in this study was extracted from unripe apples, which contain approximately 63% procyanidins, which indicates key components, including (+)-catechin, (−)-epicatechin, procyanidin B1, procyanidin B2, and procyanidin C1 [41]. In the present results, animals treated with citrate buffer alone or 2% AP showed significantly lower GST and GST-α levels compared to the normal control. Although the literature search did not provide clear evidence of direct interactions between citrate and GST isoforms, a study on cancer research reported that catechin has a strong affinity for GST-π and inhibits GST-π activity via non-competitive inhibition [42]. Catechin enriched in Applephenol^®^ may explain why it failed to enhance GST-p activity. However, whether there is an interaction between AP and GST in the context of DN that affects the measured levels requires further elucidation.

In the pathogenesis of DN, the exanimation of electron microscopy reveals specific ultrastructural changes, including GBM thickening, mesangial matrix expansion, and partial foot process effacement, underscoring the impact of DN on renal filtration function. Mitochondrial dysfunction is a critical factor driving oxidative stress in DN [43]. In this study, we observed that mitochondria in the control group exhibited normal morphology, whereas in DN tissues from diabetic rats, mitochondria demonstrated notable abnormalities in mesangial and proximal tubule cells. Administration of AP ameliorated these mitochondrial abnormalities, restoring mitochondrial morphology and reducing the number of damaged mitochondria. Previous research indicates that bavachin alleviates DN in db/db mice by inhibiting oxidative stress and enhancing mitochondrial function [38]. Additionally, green tea polyphenols have been shown to stimulate mitochondrial biogenesis and improve renal function after chronic cyclosporine A treatment in rats [44]. The interplay between ROS and mitochondria exacerbates cellular oxidative stress, which is a key factor in the progression of nephropathy. Polyphenols play a significant role in attenuating nephropathy, particularly DN, by modulating oxidative stress and promoting mitochondrial function. In the current study, RMCs were treated with H_2_O_2_, a reactive oxygen species generated under conditions of mitochondrial dysfunction and cellular oxidative stress. AP exhibited the reduction of oxidative status and TGF-β and the restoration of mitochondrial function. The use of H_2_O_2_ to treat RMC provides partial insight into the antioxidant effects of AP contributing in delaying the progression of DN. However, AP was found to reduce lipid peroxidation in kidney specimens from animals but failed to enhance GST isoform expression. This finding suggests that the biological responses to AP in RMCs and diabetic animals are not entirely consistent. In diabetic animals, sources of free radicals include advanced glycation end products, elevated lipid levels, and modified lipoproteins. Future studies should incorporate these factors into renal cell models to further elucidate the underlying mechanisms of AP. Furthermore, in addition to RMC, podocytes represent a specialized type of epithelial cell; by forming foot processes, these cells adhere tightly to GBM and play a critical role in blood filtration within the kidney. In the present study, electron microscopy analysis revealed foot process effacement in DN. Thus, selecting podocyte as a cell model represents a feasible strategy for future investigations.

TGF-β is a key mediator in DN, contributing to renal damage through mechanisms including renal cell hypertrophy, extracellular matrix accumulation, glomerulosclerosis, and interstitial fibrosis, which are further exacerbated by hyperglycemia in diabetes [45]. In this study, TGF-β expression was detected exclusively in the diabetic group at week 4, with no expression observed in the AP-treated group. By week 10, TGF-β expression was present in both diabetic and AP-treated groups; however, levels were lower in the AP-treated group, suggesting that AP may delay fibrosis associated with diabetes-induced renal damage in early DN. Research has shown that polyphenols such as resveratrol, curcumin, and apigenin reduce TGF-β expression, thereby mitigating DN [46,47,48]. Oxidative stress can induce mitochondrial damage, which in diabetic individuals may not only lead to cell death but also stimulate TGF-β expression to create a feedback loop that further exacerbates renal damage. Additionally, dyslipidemia enhances lipid peroxidation and increases ROS, and releasing can trigger TGF-β signaling to result in DN [49]. TGF-β is a prominent profibrotic protein whose increased expression and secretion in the diabetic kidney induces an epithelial-to-fibroblast transition, promoting fibrosis. While the precise mechanisms by which AP regulates TGF-β and lipid peroxidation require further investigation, our findings suggest that AP may reduce TGF-β expression, alleviate oxidative stress, and mitigate lipid peroxidation, thereby contributing to the slowing of DN progression.

In this study, Applephenon^®^ rich in procyanidins was dissolved in water and administered to animals. Previous studies indicate that grape seed procyanidins can protect renal function during the progression of DN [50]. Following oral administration, procyanidins undergo decomposition in gastric juice. Procyanidin monomers and dimers are then absorbed in the small intestine and metabolized into primary metabolites, including methylated and glucuronidated procyanidin dimers and monomers. In the colon, procyanidins are further catabolized by the colonic microflora into various low-molecular-weight phenolic acids, such as phenyl valerolactone, phenylacetic acids, and phenylpropionic acids [51]. These metabolites are subsequently modified via β-oxidation and dehydroxylation, resulting in 3- and 4-monohydroxylated phenolic acids, including vanillic acid, homovanillic acid, hippuric acid, and p-coumaric acid [52]. The glucuronidation process involves binding with glucose, which exerts a hypoglycemic effect. Phenyl valerolactone has demonstrated anti-inflammatory properties, notably inhibiting nitric oxide production [52]. Additionally, phenolic acid metabolites exhibit antioxidant, antiglycative, and antidiabetic effects. Although no hypoglycemic effect was observed in this experiment, the antioxidant and antiglycative properties of these metabolites likely reduce the production of ROS and glycated advanced end products. This reduction in oxidative and glycative stress may be a mechanism by which Applephenon^®^ components delay DN progression [53,54]. Moreover, the Applephenon^®^ used in this study was extracted from unripe apples, which indicates key components, including (+)-catechin, (−)-epicatechin, procyanidin B1, procyanidin B2, and procyanidin C1 [41]. Numerous studies have highlighted the role of individual components, such as procyanidins, in delaying the progression of DN. However, fruit extracts containing various functional components represent a more acceptable form of consumption for the general population. The polyphenol extract utilized in this study has not previously been investigated for its potential to delay DN. The current results demonstrated its beneficial effects on DN, supporting the application of natural products in diabetic patients.

Food-derived phenolics, which include structural variants of flavonoids, hydroxybenzoic acids, hydroxycinnamic acids, coumarins, stilbenes, ellagitannins, and lignans, have been shown to modulate gut microbiota composition. These compounds enhance the growth of beneficial probiotics, such as *Bifidobacterium* spp. and *E. coli*, while significantly inhibiting the growth of the *Clostridium histolyticum* group [55]. In a high-fat diet animal model, intervention with AP extract reduced the *Firmicutes*/*Bacteroidetes* ratio and increased the abundance of *Akkermansia* probiotics, which helped to prevent hepatic steatosis [56]. Through modulation of the gut microbiota, AP mitigates body weight gain induced by a high-carbohydrate diet [57], improves gene expression profiles in circulating immune cells [58], and regulates circadian rhythms in a daytime-restricted high-fat diet feeding model in C57BL/6 mice [59]. However, to date, no studies have examined the gut microbiota in relation to the effects of AP on delaying the progression of DN, highlighting an important area for future research.

Our current research has demonstrated that AP delays the progression of DN by alleviating oxidative stress; however, certain limitations remain. First, there are metabolic differences in polyphenol compounds between rats and humans. Future clinical studies are required to assess the efficacy and mechanisms of AP in delaying DN and to provide evidence applicable to human use. Second, while studies in other disease models have shown that APs can modify gut microbiota and are associated with delayed disease progression, no such studies have been conducted in DN models. Future research should include microbiota analyses to determine whether AP may delay DN through gut microbiota modulation. Third, AP can reduce lipid peroxidation in the kidney from T1DM animals but not enhance GST isoform expression. These inconsistent findings might be attributed to differences between cellular and individual responses, as well as the molecular interactions between the functional compounds in AP and GST, that need to be addressed further. Furthermore, as the average time for initial DN to reach ESRD is often cited as being around 15 to 20 years [60], 10-week administration with AP may not be sufficient for DN progression observation. Additional long-term studies and larger sample sizes are necessary to better understand the individual effect of AP and/or it synergism with other individual bioactive substances. Additionally, as procyanidin monomers and dimers are more readily absorbed in the small intestine, strategies to increase the content of these compounds within AP, thereby enhancing bioavailability, should be considered.

## 5. Conclusions

**Figure 5:**
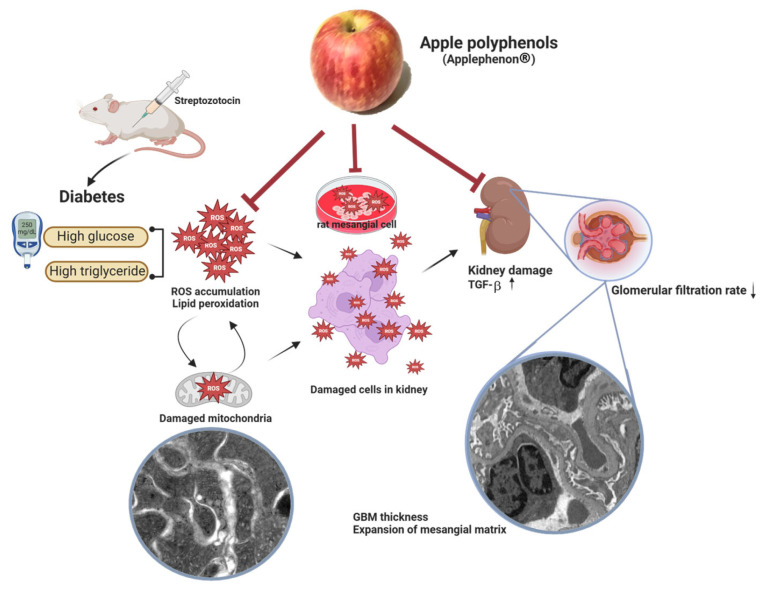
In the models of STZ-induced SD rats and H_2_O_2_-treated rat mesangial cells, AP mitigates DN by improving renal function and mitigating oxidative stress. This illustration was created in BioRender. Lee, H. (2024) http://BioRender.com/y22r012, accessed on 13 November 2024.

This study demonstrates that AP may delay the progression of DN by reducing lipid peroxidation, mitigating oxidative stress, and preserving renal function, as evidenced in both animal and cellular models (Figure 5). The antioxidative properties of AP ameliorate mitochondrial dysfunction and attenuate ultrastructural changes within renal tissues. AP reduced TGF-β expression, indicating a potential delay in fibrosis development associated with DN. This research supports AP as a promising nutraceutical candidate for managing DN, with implications for reducing the oxidative and fibrotic burden in DN. It paves a direction for clinical application of products or foods containing AP to diabetic patients, which is beneficial for medical prescriptions and daily oral intake suggestions. In the future, AP extract may be a new aspect for treating diabetic patients. Further clinical trials are warranted to make sure of its efficacy and safety.

## Figures and Tables

**Table 1:** Biochemical analysis of serum in STZ-treated Sprague–Dawley rats ^1^.

	Control	Citrate Buffer	2% AP	STZ	0.5% AP + STZ	1% AP + STZ	2% AP + STZ ^2^
Body weight (g)	545.6 ± 32 ^a^	548.4 ± 65 ^a^	544.7 ± 15 ^a^	283.2 ± 35 ^b^	286.4 ± 32 ^b^	295.3 ± 18 ^b^	343.5 ± 47 ^c^
Kidney weight (g)	3.58 ± 0.28 ^a^	3.27 ± 0.18 ^b^	3.54 ± 0.08 ^a^	3.98 ± 0.33 ^b^	3.78 ± 0.63 ^ab^	3.57 ± 0.12 ^ac^	3.46 ± 0.21 ^ac^
Glucose (mg/dL)	100.3 ± 4.3 ^a^	102.7 ± 6.2 ^a^	98.8 ± 1.7 ^a^	373.3 ± 52.9 ^b^	473.2 ± 76.5 ^b^	503.3 ± 94.1 ^b^	492.2 ± 93.6 ^b^
Blood albumin (mg/dL)	3.28 ± 0.13 ^a^	3.33 ± 0.15 ^a^	3.18 ± 0.05 ^a^	2.33 ± 0.12 ^b^	2.53 ± 0.25 ^b^	2.65 ± 0.06 ^c^	2.55 ± 0.21 ^bc^
Blood urea nitrogen (mg/dL)	15.8 ± 0.8 ^a^	15.7 ± 1.2 ^a^	13.8 ± 3.0 ^a^	41.3 ± 7.6 ^b^	59.4 ± 24.1 ^c^	51.1 ± 9.5 ^c^	40.2 ± 22.6 ^b^
Blood creatinine (mg/dL)	0.60 ± 0.0 ^a^	0.57 ± 0.1 ^a^	0.55 ± 0.1 ^a^	0.46 ± 0.1 ^b^	0.46 ± 0.1 ^b^	0.52 ± 0.1 ^c^	0.52 ± 0.1 ^c^
Blood urea nitrogen/creatinine	26.3 ± 1.3 ^a^	27.6 ± 4.0 ^a^	25.7 ± 6.4 ^a^	106.9 ± 25.3 ^b^	98.0 ± 4.8 ^b^	88.7 ± 14.8 ^b^	58.0 ± 6.6 ^c^
Urine volume (mL)	15.6 ± 1.8 ^a^	17.5 ± 4.7 ^a^	21.8 ± 7.2 ^b^	110.9 ± 38.9 ^c^	109.1 ± 22.2 ^c^	104.4 ± 21.8 ^c^	101.8 ± 29.1 ^c^
Urine microalbumin (mg/dL)	11.8 ± 3.5 ^a^	14.3 ± 10.9 ^a^	10.4 ± 0.9 ^a^	27.4 ± 6.5 ^b^	18.6 ± 3.5 ^c^	14.3 ± 2.9 ^ad^	14.2 ± 5.5 ^ad^
ACR, urine microalbumin to creatinine	0.31 ± 0.1 ^a^	0.22 ± 0.0 ^b^	0.23 ± 0.0 ^b^	3.94 ± 2.4 ^c^	1.81 ± 0.69 ^d^	1.38 ± 0.20 ^d^	1.37 ± 0.7 ^d^

^1^ The animals were treated with 65 mg/kg body weight of STZ (dissolved in 0.05 M of citrate buffer) in a single dose. After 72 h and dividing the rats with high glucose (>250 mg/dL) into a different group, the various concentrations of AP were administered for 10 weeks (*n* = 8). Three control groups were included: a normal control, a citrate buffer control (which received a single i.p. injection of 0.1 M citrate buffer), and a 2% AP-alone control. ^2^ Values (mean ± SD) not sharing a common letter in the same row are significantly different (*p* < 0.05).

**Table 2:** Effects of AP on the plasma lipid profile and the levels of antioxidative parameters in the kidneys of SD rats exposed to STZ ^1^.

	Control	Citrate Buffer	2% AP	STZ	0.5% AP + STZ	1% AP + STZ	2% AP + STZ ^2^
Total cholesterol (mg/dL)	61.0 ± 7.0 ^a^	74.7 ± 6.8 ^b^	65.3 ± 5.5 ^a^	36.0 ± 19.8 ^c^	54.0 ± 2.8 ^c^	72.3 ± 25.1 ^ab^	67.0 ± 15.1 ^ab^
Triglyceride (mg/dL)	88.5 ± 12.8 ^a^	76.0 ± 11.4 ^a^	71.0 ± 15.9 ^b^	285.5 ± 32.2 ^c^	264.0 ± 67.0 ^c^	141.3 ± 86.8 ^d^	166.7 ± 67.5 ^d^
TBARS (MDA nM/mg protein)	77.8 ± 4.4 ^a^	75.6 ± 3.6 ^a^	76.3 ± 8.9 ^a^	131.2 ± 8.7 ^b^	124.4 ± 15.2 ^b^	98.1 ± 3.9 ^c^	103.2 ± 19.8 ^c^
GST (nmole/min/mg protein)	8.0 ± 1.1 ^a^	6.4 ± 1.4 ^b^	4.9 ± 0.3 ^c^	5.3 ± 0.9 ^c^	4.6 ± 0.8 ^c^	5.4 ± 0.8 ^c^	5.2 ± 2.1 ^c^
GST-a (nmole/min/mg protein)	30.5 ± 6.5 ^a^	16.2 ± 2.7 ^b^	16.4 ± 3.1 ^b^	19.5 ± 3.2 ^c^	18.1 ± 1.7 ^c^	18.9 ± 3.1 ^c^	18.8 ± 9.5 ^bc^
GST-p (nmole/min/mg protein)	5.1 ± 0.7 ^a^	4.5 ± 0.7 ^a^	4.6 ± 0.5 ^a^	4.8 ± 0.3 ^a^	3.6 ± 0.1 ^b^	4.4 ± 0.8 ^a^	3.1 ± 0.2 ^b^

^1^ The animals were treated with 65 mg/kg body weight of STZ (dissolved in 0.05 M of citrate buffer) in a single dose. After 72 h and dividing the rats with high glucose (>250 mg/dL) into a different group, the various concentrations of AP were administered for 10 weeks (*n* = 8). Three control groups were included: a normal control, a citrate buffer control (which received a single i.p. injection of 0.1 M citrate buffer), and a 2% AP-alone control. ^2^ Values (means ± SD, *n* = 8) not sharing a common letter in the same row are significantly different (*p* < 0.05).

## Data Availability

The data used to support the findings of the study are available upon reasonable request from the corresponding author.
